# Creeping fat is associated with transmural healing in patients with Crohn’s disease receiving ustekinumab

**DOI:** 10.1186/s13244-025-02101-7

**Published:** 2025-10-04

**Authors:** Fangling Zhang, Minyi Guo, Pan Zhu, Kexin Niu, Jie Zhou, Ling Wang, Peiyi Xie, Lishuo Shi, Xiaochun Meng

**Affiliations:** 1 Department of Radiology, The Sixth Affiliated Hospital, Sun Yat-sen University, Guangzhou, China; 2 Biomedical Innovation Center, The Sixth Affiliated Hospital, Sun Yat-sen University, Guangzhou, China; 3 Center for Clinical Research, The Sixth Affiliated Hospital, Sun Yat-sen University, Guangzhou, China

**Keywords:** Crohn’s disease, Transmural healing, Ustekinumab, Creeping fat, CTE

## Abstract

**Objectives:**

We investigated whether body composition parameters assessed on baseline computed tomography enterography (CTE) could predict transmural healing (TH) in patients with Crohn’s disease (CD) receiving Ustekinumab (UST).

**Materials and methods:**

Adult patients with active CD treated with standard UST from August 2020 to August 2022 were enrolled. Body composition, including creeping fat (CF, mesenteric creeping fat index (MCFI) and fibrofatty proliferation score), skeletal muscle, visceral adipose, and subcutaneous adipose-related parameters were assessed on baseline CTE. Cox regression analysis was performed to identify independent predictors of TH.

**Results:**

This study included 113 patients, and TH occurred in 26 (23. 0%) patients. The results of the univariable analysis indicated a statistically significant association of the presence of sarcopenia, higher MCFI score, and higher fibrofatty proliferation score with an increased failure rate of TH. We found no evidence that skeletal muscle index, subcutaneous adipose index, visceral adipose index, and visceral adipose/subcutaneous adipose area ratio were associated with TH. Multivariable analysis revealed that sarcopenia (Hazard ratio (HR): 0.35, 95% CI: 0.14–0.87, *p* = 0.023), MCFI score (HR: 0.67, 95% CI: 0.49–0.91, *p* = 0.010) and fibrofatty proliferation score (HR: 0.50, 95% CI: 0.29–0.85, *p* = 0.011) remained significant. MCFI score (χ^2^-*df* = 5.58) was the most critical factor for TH prediction, followed by fibrofatty proliferation score (χ^2^-*df* = 5.43) and sarcopenia (χ^2^-*df* = 4.12).

**Conclusions:**

Among all the body composition parameters, MCFI and fibrofatty proliferation score assessed on baseline CTE were independently associated with TH, and they demonstrated greater predictive efficacy compared to sarcopenia.

**Critical relevance statement:**

Creeping fat on baseline CTE was an important predictive factor for transmural healing in patients with Crohn’s disease receiving Ustekinumab, which enables early risk stratification of patients and has potential implications for decision-making.

**Key Points:**

- Identifying predictors of transmural healing may provide insight into earlier dose optimization to improve the rate of transmural healing.
- Higher creeping fat scores (mesenteric creeping fat index and fibrofatty proliferation) were independently associated with a lower rate of transmural healing.
- Mesenteric creeping fat index and fibrofatty proliferation score demonstrated greater predictive efficacy compared to sarcopenia.

**Graphical Abstract:**

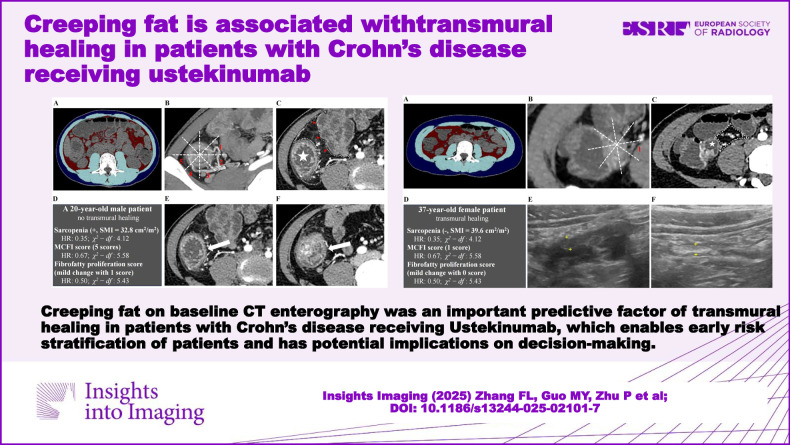

## Introduction

Crohn’s disease (CD) is a chronic, idiopathic inflammatory condition that affects the digestive tract [1]. Transmural healing (TH), characterized by the resolution of active mural inflammation and peri-intestinal abnormalities, has been recognized as a potential therapeutic goal in recent years [2]. Ustekinumab (UST) is a fully human IgG1κ monoclonal antibody targeting the p40 subunit of interleukin (IL)12/23 and has been approved for patients with moderately to severely active CD [3, 4]. Despite the effectiveness of UST in treating CD, a substantial proportion of patients are unable to achieve TH [5–8]. A major challenge in establishing TH as a formal therapeutic target is the inability to effectively achieve TH with current treatments. In this context, identifying predictors of TH is of great importance, as it can enable timely adjustments to therapeutic regimens to achieve more favorable outcomes.

Changes in body composition, characterized by a decrease in skeletal muscle mass and an increase in visceral adipose tissue (VAT) mass, have been widely observed in patients with CD. Sarcopenia, defined as a progressive decrease in skeletal muscle mass and a concurrent decline in muscle strength [9], has been found to correlate with a more severe disease phenotype and a worse prognosis [10, 11]. What’s more, there is growing evidence revealing that VAT and particularly mesenteric adipose tissue (MAT), also known as “creeping fat” (CF), contributes to the pathogenesis and progression of CD [12–16]. Consequently, recent literature has emphasized the importance of conducting body composition assessments for all individuals diagnosed with CD [17]. Quantification of skeletal muscle, VAT, and subcutaneous adipose tissue (SAT) at the third lumbar vertebra (L3) level on computed tomography (CT) is currently the most widely used approach to assess body composition. Recently, mesenteric creeping fat index (MCFI) and fibrofatty proliferation score have also been employed to evaluate the severity of CF directly and accurately on CT [18–21]. Although the negative impacts of body composition changes mentioned above on CD treatment have been studied extensively, their predictive value on TH remains poorly explored. Herein, we aimed to investigate whether body composition parameters derived from CT enterography (CTE) could predict TH in patients with CD receiving UST.

## Materials and methods

### Patients, study design, and ethics

Patients included were older than 18 years of age, confirmed with active CD based on clinical, endoscopic, radiographic, and histological data [22], and treated with standard UST strategy for more than 26 weeks during the interval from August 2020 to August 2022 consecutively. This study was conducted in compliance with the Declaration of Helsinki and was approved by the institutional ethics review board at the Sixth Affiliated Hospital, Sun Yat-sen University (No. 2023ZSLYEC). The requirement for written consent forms was waived owing to the retrospective nature.

**Fig. 1:**
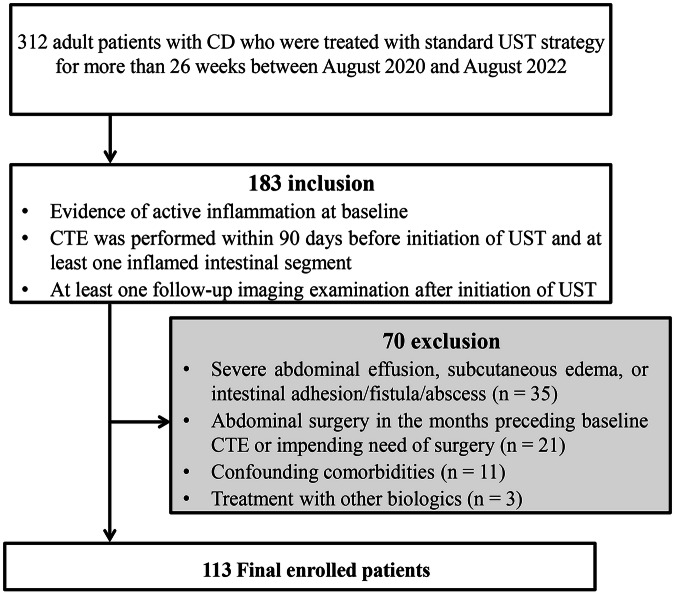
Flowchart of patient selection. CD, Crohn’s disease; UST, ustekinumab; CTE, computed tomography enterography

Figure 1 summarizes the patient selection process. The inclusion and exclusion criteria are detailed in the Supplementary Materials. Following the initiation of UST therapy, patients underwent regular follow-up every 3 or 6 months. The intestinal segment with the most severe inflammation (i.e., the most thickened) on baseline CTE was selected as the representative segment by a consensual discussion jointly by two radiologists (F.L.Z. and M.Y.G., with 10 and 8 years of experience in abdominal imaging, respectively). This segment was annotated on baseline CTE and was followed throughout the study. We divided the intestinal tract into seven segments (i.e., small intestine, terminal ileum (the intestinal segment extending proximally about 30 cm from the ileocecal valve anatomically) [23], ascending colon, transverse colon, descending colon, sigmoid colon, and rectum).

**Fig. 2:**
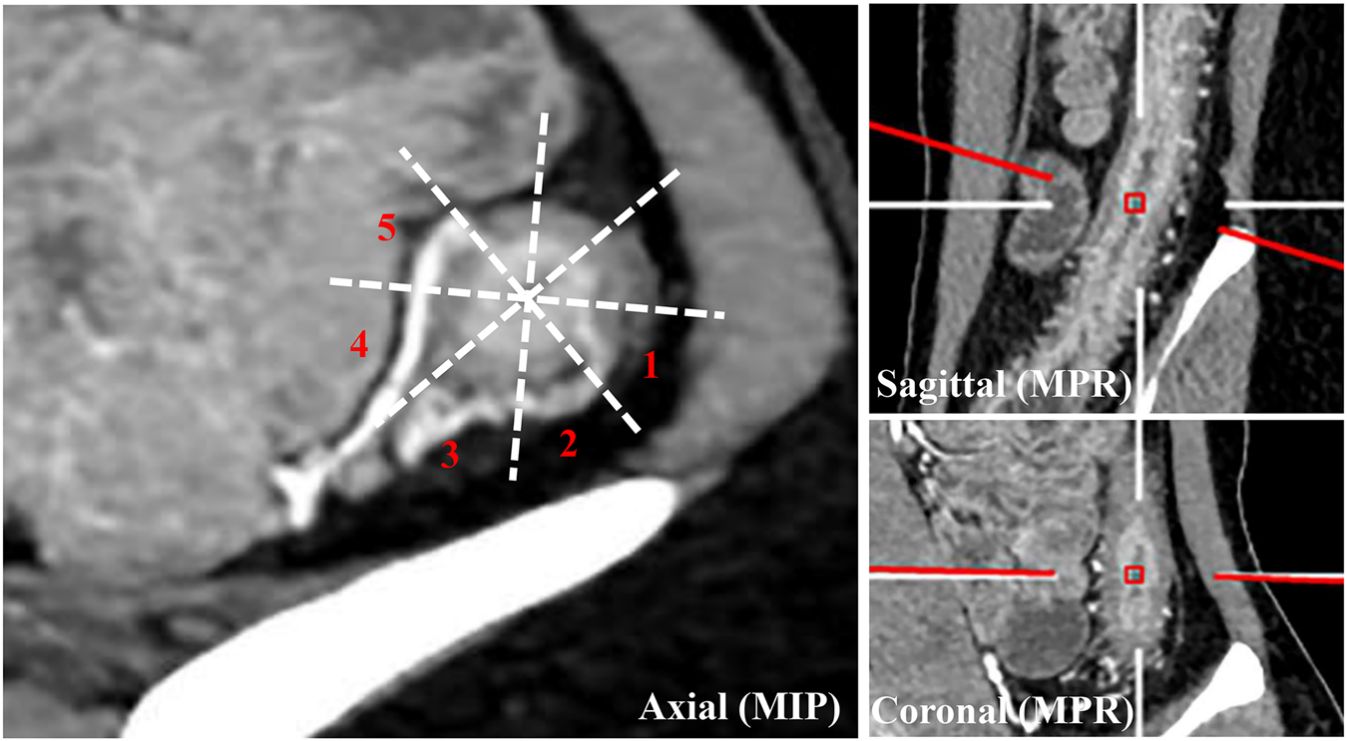
Assessment of MCFI. After orienting the slice perpendicular to the longitudinal axis of the affected segment (descending colon) using MPR (right), MIP with a slice thickness of 10 mm was applied to visualize the vessels (left). The circumference of the gut was divided into eight equal zones, and one score was assigned to each zone overlapped by vessels. The MCFI score of this patient is 5. MCFI, mesenteric creeping fat index; MPR, multiplanar reconstruction; MIP, maximum intensity projection

As outlined in Table 1 and Fig. 2, MCFI and fibrofatty proliferation score of the selected segment, along with other body composition (skeletal muscle, visceral adipose, and subcutaneous adipose-related) parameters, were assessed on baseline CTE according to literature [18, 19, 24–28]. More details regarding the body composition and the CTE protocol are shown in the Supplementary Materials. In addition, we collected baseline demographic and clinical data, which are also detailed in the Supplementary Materials.

**Table 1:** Definitions and illustrations of CTE parameters

CTE parameters	Definitions	Images		
Body composition	SMI = a / height^2^ VAI = b / height^2^ SAI = c / height^2^ VSR = b / c VTR = b / b + c	a = area of skeletal muscle	b = area of visceral adipose	c = area of subcutaneous adipose
		
MCFI	A novel radiological index grading the fat-wrapping of the inflamed intestine based on the extent to which mesenteric vessels encompass the intestinal circumference (on a scale of 1–8)	score = 2	score = 3	score = 7
		
Fibrofatty proliferation score	Increased volume of mesenteric adipose tissue around the inflamed intestine and displacement of the adjacent bowel loops (on a scale of 0–2)	score = 0	score = 1	score = 2
		

*CTE* computed tomography enterography, *SMI* skeletal muscle index, *VAI* visceral adipose index, *SAI* subcutaneous adipose index, *VSR* visceral adipose/subcutaneous adipose area ratio, *VTR* visceral adipose/total adipose area ratio, *MCFI* mesenteric creeping fat index

### Outcomes

The primary outcome of this study was TH, and it was confirmed by a consensual discussion jointly by two radiologists (F.L.Z. and M.Y.G.) who were blinded to baseline demographic and clinical data but not to baseline CTE. The confirmation of TH was based on CT, magnetic resonance imaging (MRI), or ultrasonography. If multiple examinations were conducted during the follow-up, the date of the earliest detection of TH was recorded. The follow-up endpoint was defined as the time of TH or the last follow-up for patients without TH before October 31, 2023. TH on CTE or MRE was defined as no active mural inflammation (i.e., bowel wall thickness (BWT) ≤ 3 mm, no ulceration, no edema, and no hyperenhancement), no active mesenteric sign (i.e., edema, effusion, lymphadenopathy of greater than 1 cm in short axis, and comb sign), and no complications (i.e., stricture, sinus tract, fistula, abscess or inflammatory mass) [29, 30]. TH on ultrasonography was defined as BWT ≤ 3 mm with normal blood flow signal, normalization of bowel wall stratification, and absence of inflammatory mesenteric fat [5].

### Statistical analysis

Continuous variables were expressed as either mean ± standard deviation (SD) or median ± interquartile range (IQR), depending on their distribution. Categorical variables were presented as numbers (percentages). To assess multicollinearity, the variance inflation factor (VIF) was calculated, and variables with a VIF > 10 were excluded from the subsequent analyses [31]. The Kaplan–Meier method was used to estimate the TH-free survival and cumulative incidence of TH. First, variables were analyzed using univariable Cox proportional hazard regression analysis to identify factors affecting TH. Subsequently, backward stepwise multivariable analysis was performed with variables demonstrating statistical significance (*p* < 0.05), adjusting for baseline demographic and clinical data. Results are expressed as hazard ratios (HRs) with 95% confidence intervals (CIs). The importance of each predictor was compared using the partial chi-square statistic minus the predicted degrees of freedom (χ^2^-*df*) [32]. For the interaction analysis, a product term (x × y) representing the interaction between predictor x and y was added to the multivariable Cox model to test the effect of x adjusted by y. Inter-observer agreement was assessed with the weighted kappa coefficient: < 0.2 as poor agreement, 0.2–0.4 as low, 0.4–0.6 as moderate, 0.6–0.8 as good, and > 0.8 as excellent agreement.

R statistical software (version 4.2.2, http://www.R-project.org) and SPSS (version 26.0; https://www.ibm.com/cn-zh/analytics/spss-statistics-software) were used for statistical analyses. A two-sided *p*-value of < 0.05 indicated statistical significance.

## Results

### Patients’ characteristics and CTE measurements at baseline

The final study cohort consisted of 113 patients. At baseline, CTE identified the terminal ileum as the most severely affected segment in 52 patients (46.0%), the small intestine in 29 patients (25.7%), and the colon in 32 patients (28.3%). The baseline demographic and clinical characteristics are summarized in Table 2. Notably, 17 patients (15.0%) underwent intestinal surgery previously. Regarding the medication history, 9 patients (8.0%) were medication-naïve, and 57 patients (50.4%) experienced prior exposure to at least one biologic agent. During UST treatment, 58 patients (51.3%) received concomitant medication, including corticosteroids and/or immunosuppressants. Baseline CF and other body composition parameters are presented in Table 3. The prevalence of obesity and sarcopenia was 3 patients (2.7%) and 66 patients (58.4%), respectively. Considering the collinearity among various body composition parameters, the visceral adipose/total adipose area ratio (VTR) was excluded (VIF = 13.02). Finally, the following parameters were included in the subsequent analyses: baseline MCFI, fibrofatty proliferation score, SMI, sarcopenia, SAI, VAI, and VSR, all of which had VIF values ranging from 1.09 to 9.84. The median MCFI score was 4 (IQR, 3–5), and the median fibrofatty proliferation score was 1 (IQR, 1–2). Inter-observer agreement between the two radiologists was good for the MCFI score (weighted kappa = 0.798) and excellent for the fibrofatty proliferation score (weighted kappa = 0.851).

**Table 2:** Baseline demographic and clinical characteristics in patients with CD

Characteristic	*n* = 113
Gender, *n* (%)	
Male	84 (74.3﻿)
Female	29 (25.7﻿)
Age, *n* (%)	
≤ 40	94 (83.2)
> 40	19 (16.8)
Smoking history, *n* (%)	18 (15.9)
CD duration (years), median (IQR)	1.0 (1.0, 5.0)
Montreal classification	
CD location, *n* (%)	
L1: Ileal	34 (30.1)
L2: Colonic	5 (4.4)
L3: leocolonic	74 (65.5)
L4:Upper gastrointestinal tract involvement	6 (5.3)
CD behavior, *n* (%)	
B1: Inflammatory	66 (58.4)
B2: Stricturing	38 (33.6)
B3: Penetrating	9 (8.0)
Perianal lesion	57 (50.4)
Prior bowel resection, *n* (%)	17 (15.0)
Medication history, *n* (%)	
Medication naïve	9 (8.0)
Prior biologic exposure	
0	56 (49.6)
1	43 (38.1)
2	12 (10.6)
3	2 (1.8)
CRP (mg/L)	
Median (IQR)	9.6 (3.4, 28.6)
Abnormal CRP^a^, *n* (%)	72 (63.7)
ESR (mm/h), median (IQR)	17.0 (8.0, 35.5)
Concomitant medication of UST, *n* (%)	58 (51.3)
Interval between CTE and UST initiation (days), median (IQR)	5.0 (2.0, 21.0)

*CD* Crohn’s disease, *IQR* interquartile range, *CRP* C-reactive protein, *ESR* erythrocyte sedimentation rate, *UST* ustekinumab, *CTE* computed tomography enterography

Continuous variables were presented as mean ± standard deviation (SD) or median (interquartile range, IQR) depending on the distribution of the data. Categorical data were expressed as numbers (percentages)

^a^ Abnormal CRP concentration was >  5 mg/L

**Table 3:** Baseline MCFI, fibrofatty proliferation score and other body composition parameters in patients with CD

Parameter	*n* = 113
Obesity^a^, *n* (%)	3 (2.7)
SMI, median (IQR)	40.0 (33.6, 44.6)
Sarcopenia, *n* (%)	66 (58.4)
VAI, median (IQR)	12.8 (7.0, 24.6)
SAI, median (IQR)	22.6 (11.2, 36.7)
VSR, median (IQR)	0.6 (0.7, 1.1)
MCFI, median (IQR)	4 (3, 5)
1, *n* (%)	2 (1.8)
2, *n* (%)	14 (12.4)
3, *n* (%)	24 (21.2)
4, *n* (%)	25 (22.1)
5, *n* (%)	21 (18.6)
6, *n* (%)	18 (15.9)
7, *n* (%)	8 (7.1)
8, *n* (%)	1 (0.9)
Fibrofatty proliferation score, median (IQR)	1 (1, 2)
0, *n* (%)	27 (23.9)
1, *n* (%)	45 (39.8)
2, *n* (%)	41 (36.3)

*MCFI* mesenteric creeping fat index, *CD* Crohn’s disease, *SMI* skeletal muscle index, *IQR* interquartile range, *VAI* visceral adipose index, *SAI* subcutaneous adipose index, *VSR* visceral adipose/subcutaneous adipose area ratio

Continuous variables were presented as mean ± standard deviation (SD) or median (interquartile range, IQR) depending on the distribution of the data. Categorical data were expressed as numbers (percentages)

^a^ Body mass index (BMI) > 30 kg/m^2^ was obesity

### Prediction of TH

During the observation period, the outcome of TH occurred in 26 patients (23. 0%) within the entire study population. The median follow-up period for TH is 61.0 weeks (95% CI: 56.1–65.9). The median TH-free survival was 112.0 weeks (95% CI: 76.1–147.9). The cumulative incidence of TH at 26 weeks, 52 weeks, and 104 weeks was 7.1% (95% CI: 5.1, 10.3), 14.2% (95% CI: 14.1, 22.1), and 22.1% (95% CI: 0.527, 0.695), respectively.

**Fig. 3:**
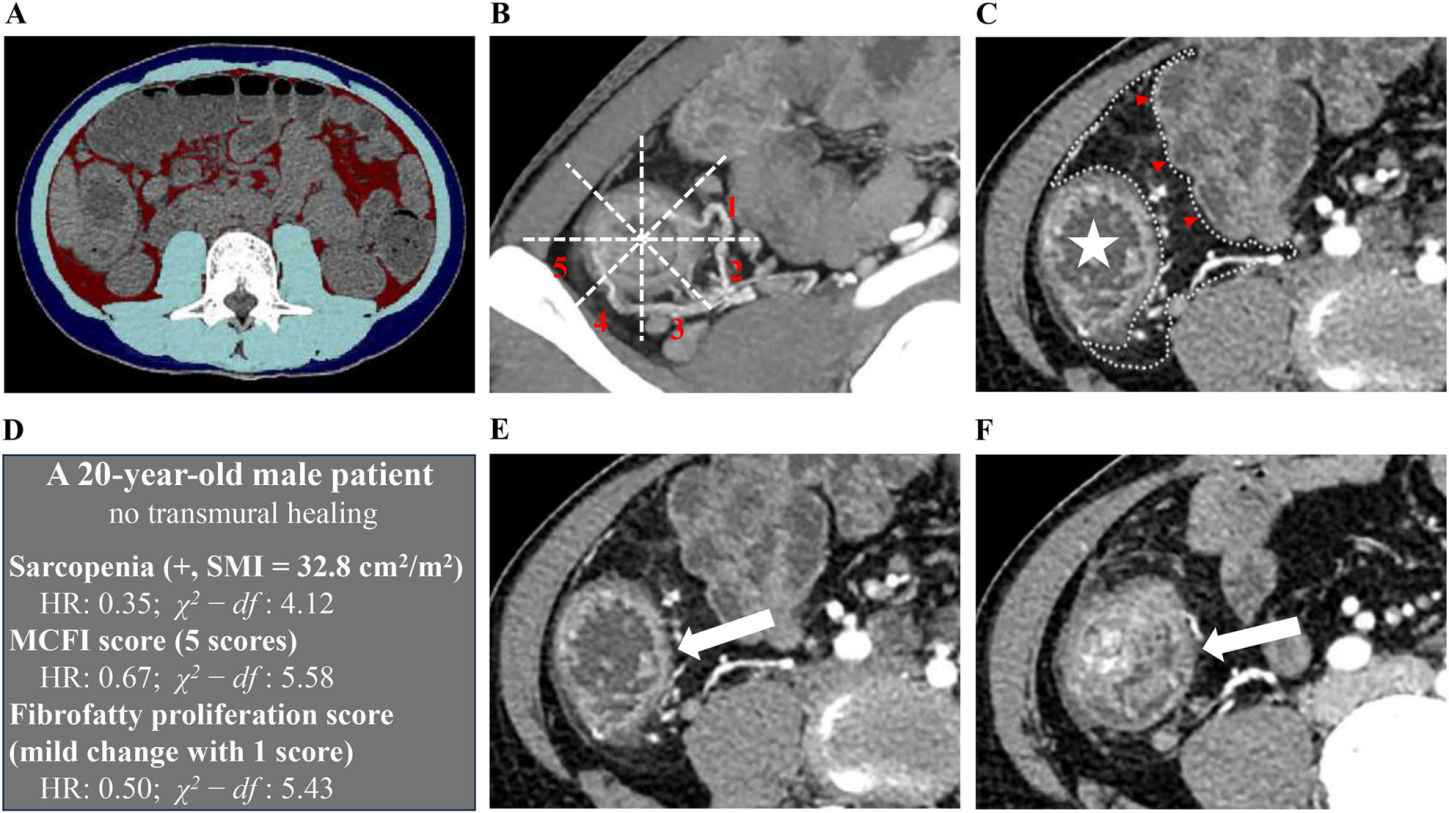
A patient who experienced a failure to achieve TH (**A**–**F**). **A** Axial non-contrast CT image at the L3 level shows the segmentation of skeletal muscle (light blue), visceral adipose tissue (red), and subcutaneous adipose tissue (dark blue). The patient was diagnosed with sarcopenia, with an SMI of 32.8 cm^2^/m^2^. **B**, **C** The MCFI and fibrofatty proliferation score of the ascending colon (indicated by the star) are scored as 5 and 1, respectively. **E** Axial post-contrast CT image shows the pretreatment inflammation of the ascending colon (arrow). **F** After 74 weeks of UST treatment, this patient experienced a failure to achieve TH, with BWT > 3 mm, mural hyperenhancement, and mesenteric edema. TH, transmural healing; L3, third lumbar vertebra; MCFI, mesenteric creeping fat index; SMI, skeletal muscle index; UST, ustekinumab; BWT, bowel wall thickness

**Fig. 4:**
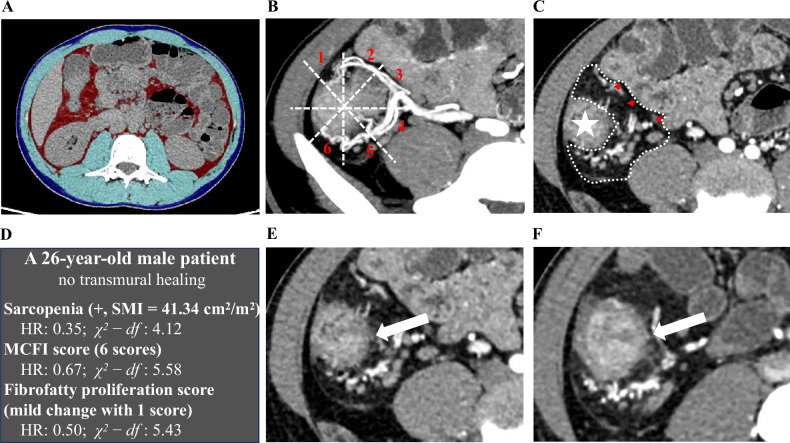
Another patient who experienced a failure to achieve TH (**A**–**F**). **A** Axial non-contrast CT image at the L3 level shows the segmentation of skeletal muscle (light blue), visceral adipose tissue (red), and subcutaneous adipose tissue (dark blue). The patient was diagnosed with sarcopenia, with an SMI of 41.34 cm^2^/m^2^. **B**, **C** The MCFI and fibrofatty proliferation score of the ascending colon (indicated by the star) are scored as 6 and 1, respectively. **E** Axial post-contrast CT image shows the pretreatment inflammation of the ascending colon (arrow). **F** After 62 weeks of UST treatment, this patient experienced a failure to achieve TH, with BWT > 3 mm, mural hyperenhancement, and mesenteric edema. TH, transmural healing; L3, third lumbar vertebra; MCFI, mesenteric creeping fat index; SMI, skeletal muscle index; UST, ustekinumab; BWT, bowel wall thickness

**Fig. 5:**
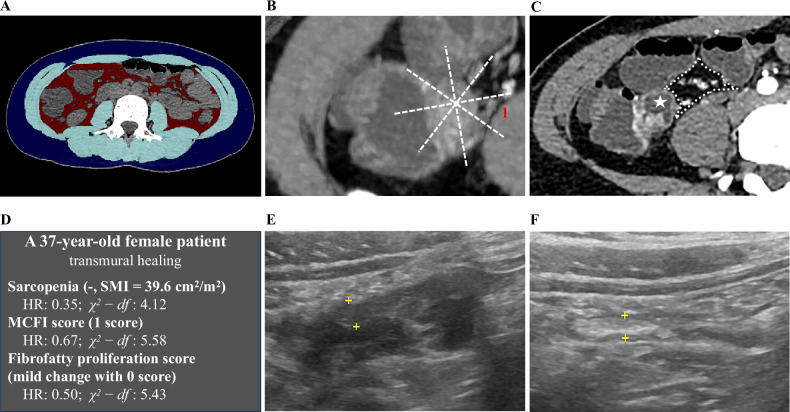
A patient who achieved TH (**A**–**F**). **A** Axial non-contrast CT image at the L3 level shows the segmentation of skeletal muscle (light blue), visceral adipose tissue (red), and subcutaneous adipose tissue (dark blue). The patient was diagnosed with no sarcopenia, with an SMI of 39.6 cm^2^/m^2^. **B**, **C** The MCFI and fibrofatty proliferation score of the terminal ileum (indicated by the star) are scored as 1 and 0, respectively. **E** Pretreatment ultrasonography shows inflammation of the terminal ileum (plus sign). **F** After 60 weeks of UST treatment, this patient achieved TH, with BWT ≤ 3 mm, normalization of blood flow signal, absence of bowel wall stratification, and no inflammatory mesenteric fat. TH, transmural healing; L3, third lumbar vertebra; MCFI, mesenteric creeping fat index; SMI, skeletal muscle index; UST, ustekinumab; BWT, bowel wall thickness

The results of the univariable Cox regression analysis indicated a statistically significant association of the presence of sarcopenia (*p* = 0.035), higher MCFI score (*p* = 0.037), and higher fibrofatty proliferation score (*p* = 0.026) with an increased failure rate of TH. No evidence was found that SMI (*p* = 0.909), SAI (*p* = 0.225), VAI (*p* = 0.250), VSR (*p* = 0.404), and BMI (*p* = 0.218) were associated with TH. When VAI and VSR were treated as binary variables according to the median value, there was still no statistically significant association with TH (*p* = 0.307 and *p* = 0.154, respectively). Multivariable analysis revealed that sarcopenia (HR: 0.35, 95% CI: 0.14–0.87; *p* = 0.023), MCFI score (HR: 0.67, 95% CI: 0.49–0.91; *p* = 0.010) and fibrofatty proliferation score (HR: 0.50, 95% CI: 0.29–0.85; *p* = 0.011) remained significant after adjusting for potential confounders, as shown in Table 4. MCFI score (χ^2^-*df* = 5.58) was found to be the most critical factor for TH prediction, followed by fibrofatty proliferation score (χ^2^-*df* = 5.43) and sarcopenia (χ^2^-*df* = 4.12). Figures 3–5 display three patients with different outcomes.

**Table 4:** Univariable and multivariable Cox regression analyses for transmural healing (*n* = 113)

Variable	Univariable analysis		Multivariable analysis	
	HR (95% CI)	*p*-value	HR (95% CI)	*p*-value
BMI	3.57 (0.47, 26.87)	0.218		
SMI	1.00 (0.99, 1.01)	0.909		
Sarcopenia	0.42 (0.19, 0.94)	**0.035**	0.35 (0.14, 0.87)	**0.023**
SAI	0.98 (0.96, 1.01)	0.225		
VAI	0.98 (0.94, 1.02)	0.250		
VSR	0.67 (0.26, 1.72)	0.404		
MCFI	0.75 (0.58, 0.98)	**0.037**	0.67 (0.49, 0.91)	**0.010**
Fibrofatty proliferation score	0.54 (0.32, 0.93)	**0.026**	0.50 (0.29, 0.85)	**0.011**
CRP	—	—	3.95 (1.38, 11.28)	**0.010**
Medication naïve	—	—	4.04 (1.15, 14.13)	**0.029**

Data are hazard ratios (HRs), with 95% confidence intervals (CIs) in parentheses. “—” indicates not available

*BMI* body mass index, *SMI* skeletal muscle index, *SAI* subcutaneous adipose index, *VAI* visceral adipose index, *VSR* visceral to subcutaneous adipose area ratio, *MCFI* mesenteric creeping fat index, *CRP* C-reactive protein

Bold values indicated statistical significance (*p*-value ＜ 0.05)

### Interaction analysis

In the interaction analysis, the MCFI score-by-sarcopenia (*p* = 0.951), MCFI score-by-fibrofatty proliferation score (*p* = 0.462), and fibrofatty proliferation score-by-sarcopenia (*p* = 0.860) interactions were nonsignificant for TH in the adjusted model. Additionally, sarcopenia (*p* = 0.837), MCFI score (*p* = 0.306), and fibrofatty proliferation score (*p* = 0.862) did not significantly interact with sex for TH, respectively. Similarly, nonsignificant results were observed for other baseline demographic and clinical data.

### Sensitivity analysis

As insufficient follow-up may have a certain impact on the study results, sensitivity analysis was performed after exclusion of individuals with less than 26 weeks of follow-up (*n* = 16), which can generate a new population to verify the robustness of our findings. The results of the sensitivity analysis showed that our results were robust. Univariable Cox regression analysis showed that patients with sarcopenia (*p* = 0.041), higher MCFI score (*p* = 0.026) and higher fibrofatty proliferation score (*p* = 0.037) were less likely to achieve TH. Subsequently, multivariable analysis revealed that sarcopenia (HR: 0.35, 95% CI: 0.14–0.87; *p* = 0.024), MCFI score (HR: 0.66, 95% CI: 0.49–0.91; *p* = 0.010) and fibrofatty proliferation score (HR: 0.51, 95% CI: 0.30–0.87; *p* = 0.013) were independently associated with TH after adjusting for baseline demographic and clinical factors (Table S1).

## Discussion

Our study explored different body composition parameters obtained from baseline CTE and successfully identified predictors for TH in patients with CD receiving UST. The data demonstrated that patients with sarcopenia were less likely to achieve TH. Although no evidence of an association between VAI, VSR, and TH was found, patients with higher MCFI and fibrofatty proliferation scores were associated with a decreased rate of TH. In addition, MCFI and fibrofatty proliferation score demonstrated greater predictive efficacy compared to sarcopenia.

Patients with CD are at a high risk of sarcopenia due to malnutrition, immune, and metabolic disturbances [33, 34]. A recent systematic review concluded that sarcopenia was highly prevalent in the inflammatory bowel disease (IBD) population, affecting 43% of patients, even among those with unchanged or elevated BMI [35]. Despite the detrimental effect of sarcopenia being demonstrated in a wide variety of studies [35–40], data on the association between sarcopenia and TH in patients with CD receiving UST are scarce. The prevalence of sarcopenia in our study was 58.4%, and sarcopenia correlated with a decreased rate of TH, which is consistent with existing findings. Currently, the mechanism by which sarcopenia affects the efficacy of drug treatment remains unknown. It may be related to reduced drug absorption [41–43]. Pretreatment sarcopenia screening, facilitated by the accurate and reproducible measure of skeletal muscle on CTE, provides an approach for the prediction of TH. Patients may benefit from treatment aimed at reversing low muscle mass, such as nutritional interventions and exercise [44].

Considering different adipose tissue compartments, it appears that VAT, especially hyperplastic MAT or CF, has crucial clinical implications on CD [45–48]. Despite progress that has been made in recent years, the formation mechanism and the role of CF remain obscure. As a result of impaired epithelial integrity, intestinal microbes translocate into the mesentery. The interaction of adipocytes with microbes induces adipocyte hyperplasia, immunoreaction, and the secretion of proinflammatory cytokines, which in turn results in a “second hit” to the adjacent intestinal wall [49]. An exploratory study aiming to investigate CF as a new therapeutic target found that CF was reversible under certain conditions, and this process was accompanied by a certain degree of inflammation alleviation [50]. This further supports the proinflammatory potential of CF and its negative impact on CD prognosis. It is now generally believed that CF is linked to active inflammation because it can be present as early as the disease onset [51]. As our previous findings revealed, CF was a risk factor for adverse prognosis in patients with endoscopic healing [52], and it also serves as an indicator of chronic and persistent intestinal damage during the remission phase. In addition, the impacts of CF on the efficacy of other drugs have been widely confirmed [53, 54]. These findings suggest CF may be a powerful predictor of TH in patients with CD receiving UST. Since VAT-related parameters (VAI, VSR), MCFI, and fibrofatty proliferation score on CTE have been used to quantify CF in the literature, we included them in our analyses.

In contrast to most current studies, our results did not find any significant correlation between VAT-related parameters and TH, although these ratios may be valuable in reflecting the quantity of visceral adipose and changes in fat distribution. This can be interpreted because VAT mass was overestimated on CTE, as retroperitoneal adipose was classified as VAT. In addition, although CF in CD can lead to an increase in visceral fat, as an overall metric, visceral fat also includes mesenteric fat surrounding normal intestinal segments, which varies greatly among individuals. Several previous studies were consistent with our perspective [55, 56]. However, the vast majority of studies have focused on VAT rather than CF itself as a research target so far. Therefore, future fat-related studies of CD should focus on the localized CF in specific intestinal segments rather than the overall visceral fat. As expected, MCFI and fibrofatty proliferation score were independent predictors of TH in our study. This is likely because these metrics specifically targeted CF. Our study sheds light on the role of MCFI in predicting TH, thereby adding to the existing body of knowledge in this area [18, 21]. Before the development of MCFI, fibrofatty proliferation had been utilized in several studies involving cross-sectional imaging [13, 54]. In our study, a significant decrease in the possibility of TH was found in patients with high fibrofatty proliferation scores, and the predictive performance was comparable to that of MCFI, which is consistent with previous studies.

Some limitations should be mentioned. First, there is a lack of a standardized and widely accepted definition of TH to date, which restricts the generalizability of our findings. Second, the sample size of our study cohort was limited. Third, this study was retrospective, which inevitably introduces selection bias and prevents the inclusion of certain potential factors such as fecal calprotectin, drug levels, and antibodies. Fourth, our study only focused on the intestinal segment with the most severe inflammation because the less involved segments commonly respond in parallel to the most affected segment in clinical practice. To enhance the rigor of the study design, we should evaluate all inflamed segments. Lastly, we did not classify the data into “small bowel and colon groups” as many studies have suggested that small bowel and colon CD represent two distinct disease entities [57, 58].

In conclusion, MCFI and fibrofatty proliferation score on baseline CTE were important predictive factors of TH in patients with CD receiving UST. These metrics demonstrated greater predictive efficacy compared to sarcopenia, enabling early risk stratification of patients and offering potential implications for decision-making.

## Supplementary information

ELECTRONIC SUPPLEMENTARY MATERIAL

## Data Availability

The datasets used and/or analyzed during the current study are available from the corresponding author upon reasonable request.

## Abbreviations

BWT: Bowel wall thickness
CD: Crohn’s disease
CF: Creeping fat
CI: Confidence interval
CT: Computed tomography
CTE: CT enterography
HR: Hazard ratio
IQR: Interquartile range
L3: Third lumbar vertebra
MAT: Mesenteric adipose tissue
MCFI: Mesenteric creeping fat index
SD: Standard deviation
TH: Transmural healing
UST: Ustekinumab
VAT: Visceral adipose tissue
VIF: Variance inflation factor
VTR: Visceral adipose/total adipose area ratio
χ^2^-*df*: Chi-square statistic minus the predicted degrees of freedom
