# Supplementary material for: Creeping fat is associated with transmural healing in patients with Crohn’s disease receiving ustekinumab
Source: Insights Imaging. 2025 Oct 4;16:214. doi: 10.1186/s13244-025-02101-7 (PMC12496395; doi:10.1186/s13244-025-02101-7)
Supplement: Supplementary file 1 — ELECTRONIC SUPPLEMENTARY MATERIAL [file 13244_2025_2101_MOESM1_ESM.pdf]

Creeping fat is associated with transmural healing in patients with Crohn's disease receiving Ustekinumab

ELECTRONIC SUPPLEMENTARY MATERIAL

METHODS

Inclusion and exclusion criteria

The inclusion criteria were as follows:

(1) Computed tomography enterography (CTE) was performed at baseline within 90 days before the initiation of UST, and there was at least one intestinal segment with active inflammation (i.e., bowel wall thickness [BWT]＞3 mm and mural hyperenhancement)

(2) At least one follow-up imaging examination was conducted after the initiation of Ustekinumab (UST).

Patients were excluded if:

(1) The contours of the bowel wall, visceral adipose tissue (VAT), subcutaneous adipose tissue (SAT), and designated mesenteric adipose tissue (MAT) were not readily identifiable on CTE due to severe abdominal effusion, subcutaneous edema, intestinal adhesion/fistula/abscess, or poor CTE imaging quality;

(2) They underwent abdominal surgery in the months preceding baseline CTE or were in impending need of surgery due to Crohn's disease (CD)-related complications;

(3) They were diagnosed with confounding comorbidities, such as metabolic diseases, immunological diseases, cancer, or severe organ insufficiency;

(4) They were treated with other biologics (e.g., infliximab, adalimumab, vedolizumab, and certolizumab) during UST therapy.

CTE protocol

CTE scans were acquired by Aquilion Vision (Canon Medical Systems, Otawara, Japan) at our institution. All patients fasted for at least 6 hours before the CTE scans. A total of 1500–2000 mL of 2.5% mannitol solution was administered orally 45-60 minutes before image acquisition to ensure adequate distension of the gut. To avoid gastrointestinal peristalsis, an intramuscular injection of 10 mg racemic anisodamine hydrochloride was administered 10-15 minutes before CTE examination. After non-contrast scanning, non-ionic contrast media (Ultravist 370; Schering, Berlin, Germany) at a dosage of 2 mL per kilogram was injected intravenously at a rate of 3.5 mL/s using a dual-head power injector, followed by an immediate saline flush. All patients underwent enteric phase and venous phase imaging, which started 45 seconds and 70 seconds after injection, respectively. CT examinations were performed during a breath-hold, covering the range from the dome of the liver to the symphysis pubis in the supine position. The scan parameters were as follows: Tube voltage: 120 kVp, Tube current: automatic mA, Pitch: 0.813, Slice thickness: 1mm, Reconstruction interval: 1mm. The technology of adaptive iterative dose reduction was adopted to reduce radiation dose.

Assessment of creeping fat (CF) and other body composition parameters

Referring to a previous study[1], the mesenteric creeping fat index (MCFI) is a novel radiological index used to grade CF around the inflamed intestine based on the extent to which mesenteric vessels encompass the bowel circumference. After orienting the slice perpendicular to the longitudinal axis of the affected segment using multiplanar reconstruction (MPR), maximum intensity projection (MIP) was applied to visualize the vessels. The circumference of the bowel was partitioned into eight equal zones, with each zone overlapping with vessels assigned one score. A higher cumulative score indicated more severe CF, as outlined in Table 1 and Fig.2. As another index for semi-quantifying CF radiographically, the fibrofatty proliferation score considered

the volume of MAT around the inflamed intestine and the displacement of the adjacent bowel loops[1-4]. A score of 0 indicates the absence of changes, 1 indicates mild changes, and 2 indicates moderate or severe changes (Table 1). Two radiologists (F.L.Z. and M.Y.G., with 10 and 8 years of experience in abdominal imaging, respectively), who were unaware of outcome information, independently measured MCFI and fibrofatty proliferation score of the selected segments. Disagreements were resolved by discussion until a final consensus was reached.

A single axial slice from the non-contrast CTE scan at the middle level of the L3 vertebral body was selected to measure the area of skeletal muscle, VAT, and SAT. The process of semi-automated segmentation and quantification was conducted by a trained radiologist (Z.F.L.) who was blinded to clinical data and outcomes, using Slice Omatic 5.0 (TomoVision, Montreal, Canada). The segmentation employed tissue-specific attenuation thresholds, with ranges of -29 to 150 HU for skeletal muscle, -150 to -50 HU for VAT, and -190 to -30 HU for SAT, consistent with findings from prior research[5-7]. Skeletal muscle comprises the psoas major, erector spinae, quadratus lumborum, transversus abdominis, external oblique, internal oblique, and rectus abdominis. Additional manual corrections were made according to tissue planes, and a final review of the segmentations was performed by a senior radiologist (X.C.M., with more than 20 years of experience in abdominal imaging). Further standardized indices, including the skeletal muscle index (SMI), visceral adipose index (VAI), and subcutaneous adipose index (SAI), were calculated using a formula: area (in square centimeters [cm^2])/height squared (in square meters [m^2]). According to previously reported criteria, sarcopenia was defined as SMI < 32.5 cm^2/m^2 for females and < 44.77 cm^2/m^2 for males, respectively[8]. The VAT/SAT ratio (VSR) and the visceral adipose / total adipose area ratio (VTR) were calculated to reflect abdominal fat distribution. An example of the segmentation is shown in Table 1.

Baseline demographic and clinical data collection

Baseline demographic and clinical data within one week before UST treatment were extracted from the electronic medical records by a single radiologist (K.X.N., with 5 years of experience in abdominal imaging) who was blinded to the CTE measurements and outcome information. We recorded the following variables: sex, age, smoking history, body mass index (BMI), disease duration, Montreal classification, previous bowel resection, medication history (medication-naïve and prior biological exposure), concomitant medication of UST, C-reactive protein (CRP), and erythrocyte sedimentation rate (ESR) levels. Medication-naïve was defined as never previous use of infliximab, adalimumab, vedolizumab, or certolizumab. Concomitant medication included systemic corticosteroids, budesonide, and immunosuppressants during UST therapy.

Table S1. Univariable and multivariable Cox regression analyses for transmural healing (n=97)

Variable	Univariable Analysis		Multivariable Analysis	
	HR (95% CI)	*P* Value	HR (95% CI)	*P* Value
**BMI**	3.50 (0.47, 26.42)	0.224		
**SMI**	1.00 (0.99, 1.01)	0.944		
**Sarcopenia**	0.43 (0.19, 0.97)	**0.041**	0.35 (0.14, 0.87)	**0.024**
**SAI**	0.99 (0.96, 1.01)	0.271		
**VAI**	0.98 (0.94, 1.02)	0.271		
**VSR**	0.65 (0.25, 1.68)	0.374		
**MCFI**	0.74 (0.56, 0.96)	**0.026**	0.66 (0.49, 0.91)	**0.010**
**Fibrofatty proliferation score**	0.56 (0.33, 0.97)	**0.037**	0.51 (0.30, 0.87)	**0.013**
**CRP**	—	—	4.01 (1.38, 11.67)	**0.011**
**Medication naïve**	—	—	4.30 (1.21, 15.33)	**0.025**

Table S1. Univariable and multivariable Cox regression analyses for transmural healing (n=97)

Data are presented as hazard ratios(HRs), with 95% confidence intervals (CIs) in parentheses.

— Not available.

BMI body mass index, SMI Skeletal muscle index, SAI Subcutaneous adipose index, VAI Visceral adipose index, VSR visceral to subcutaneous adipose area ratio, MCFI mesenteric creeping fat index, CRP C-reactive protein.

References

1. Li XH, Feng ST, Cao QH, et al. Degree of Creeping Fat Assessed by Computed Tomography Enterography is Associated with Intestinal Fibrotic Stricture in Patients with Crohn's Disease: A Potentially Novel Mesenteric Creeping Fat Index. J Crohns Colitis 2021. 15(7):1161-1173.

2. Rimola J, Alfaro I, Fernández-Clotet A, et al. Persistent damage on magnetic resonance enterography in patients with Crohn's disease in endoscopic remission. Aliment Pharmacol Ther 2018. 48(11-12):1232-1241.

3. Koh DM, Miao Y, Chinn RJ, et al. MR imaging evaluation of the activity of Crohn's disease. AJR Am J Roentgenol 2001. 177(6):1325-1332.

4. Sakurai T, Katsuno T, Saito K, et al. Mesenteric findings of CT enterography are well correlated with the endoscopic severity of Crohn's disease. Eur J Radiol 2017. 89:242-248.

5. Mitsiopoulos N, Baumgartner RN, Heymsfield SB, et al. Cadaver validation of skeletal muscle measurement by magnetic resonance imaging and computerized tomography. J Appl Physiol (1985) 1998. 85(1):115-122.

6. Mourtzakis M, Prado CM, Lieffers JR, et al. A practical and precise approach to quantification of body composition in cancer patients using computed tomography images acquired during routine care. Appl Physiol Nutr Metab 2008. 33(5):997-1006.

7. Yoshizumi T, Nakamura T, Yamane M, et al. Abdominal fat: standardized technique for measurement at CT. Radiology 1999. 211(1):283-286.

8. Zeng X, Shi ZW, Yu JJ, et al. Sarcopenia as a prognostic predictor of liver cirrhosis: a multicentre study in China. J Cachexia Sarcopenia Muscle 2021. 12(6):1948-1958.
